# Detection of autoantibodies to DSF70/LEDGFp75 in Mexican Hispanics using multiple complementary assay platforms

**DOI:** 10.1007/s13317-016-0089-7

**Published:** 2016-11-24

**Authors:** Mónica Vázquez-Del Mercado, Eduardo Gómez-Bañuelos, Rosa Elena Navarro-Hernández, Oscar Pizano-Martinez, Adan Saldaña-Millán, Efrain Chavarria-Avila, Lorena Gonzalez-Rosas, Lilia Andrade-Ortega, Miguel Angel Saavedra, Olga Lidia Vera-Lastra, Luis Javier Jara, Gabriel Medrano-Ramírez, Claudia Cruz-Reyes, Ignacio García-De la Torre, Marta Escarra-Senmarti, Lisiane Maria Enriconi-Dos Anjos, Anamika Basu, Roger Albesa, Michael Mahler, Carlos A. Casiano

**Affiliations:** 1 Instituto de Investigación en Reumatología y del Sistema Músculo Esquelético, CUCS, Universidad de Guadalajara, Guadalajara, Jalisco Mexico; 2 Servicio de Reumatología, Posgrado 004086 PNPC-CONACyT, División de Medicina Interna, O.P.D., Hospital Civil Dr. Juan I. Menchaca, Guadalajara, Jalisco Mexico; 3 Servicio de Reumatología, Centro Médico Nacional 20 de Noviembre ISSSTE, Mexico, D.F. Mexico; 4 Hospital de Especialidades, Centro Médico Nacional La Raza, IMSS, Mexico, D.F. Mexico; 5 Servicio de Reumatología, Hospital General de México, Mexico, D.F. Mexico; 6 Servicio de Inmunología y Reumatología, Hospital General de Occidente, Zapopan, Jalisco Mexico; 7 Department of Basic Sciences, Center for Health Disparities and Molecular Medicine, Loma Linda University School of Medicine, Mortensen Hall 142, 11085 Campus St., Loma Linda, CA 92350 USA; 8 Department of Research, Inova Diagnostics, Inc., San Diego, CA USA; 9 Department of Medicine, Division of Rheumatology, Loma Linda University School of Medicine, Loma Linda, CA USA

**Keywords:** DFS70, LEDGF/p75, Autoantibodies, Frequency, Mexican, Hispanics, Multiple assay platforms

## Abstract

**Purpose:**

Antinuclear autoantibodies (ANA) targeting the dense fine speckled antigen DFS70, also known as lens epithelium-derived growth factor p75 (LEDGF/p75), are attracting attention due to their low frequency in systemic rheumatic diseases but increased frequency in clinical laboratory referrals and healthy individuals (HI). These ANA specifically recognize the stress protein DFS70/LEDGFp75, implicated in cancer, HIV-AIDS, and inflammation. While their frequency has been investigated in various ethnic populations, there is little information on their frequency among Hispanics/Latinos. In this study, we determined the frequency of anti-DFS70/LEDGFp75 autoantibodies in Mexican Hispanics using multiple detection platforms.

**Methods:**

The frequency of anti-DFS70/LEDGFp75 antibodies was determined in 171 individuals, including 71 dermatomyositis (DM) patients, 47 rheumatoid arthritis (RA) patients, 30 obesity (OB) patients, and 23 HI. Antibody detection was achieved using four complementary assay platforms: indirect immunofluorescence, Western blotting, ELISA, and chemiluminescent immunoassay.

**Results:**

We detected relatively low frequencies of anti-DFS70/LEDGFp75 antibodies in patients with DM (1.4%), RA (4.3%), and OB (6.6%), and elevated frequency (17.4%) in HI. A strong concordance between the different antibody detection platforms was observed.

**Conclusions:**

The low frequency of anti-DFS70/LEDGFp75 antibodies in Mexican patients with rheumatic diseases, but relatively higher frequency in HI, is consistent with previous observations with non-Hispanic populations, suggesting that geographic differences or ethnicity do not influence the frequency of these autoantibodies. Our results also highlight the importance of confirmatory assays for the accurate detection of these autoantibodies. Future studies with larger cohorts of healthy Hispanics/Latinos are needed to confirm if their anti-DFS70/LEDGFp75 antibody frequencies are significantly higher than in non-Hispanics.

## Introduction

The presence of antinuclear autoantibodies (ANA) is a key feature of ANA-associated rheumatic diseases (AARD) such as systemic lupus erythematosus and scleroderma [1]. The dense fine speckled (DFS) ANA pattern has recently been the subject of intense investigation since it is one the most commonly recognized autoantibody patterns produced by human sera referred to clinical laboratories for ANA testing by indirect immunofluorescence (IIF) microscopy in HEp-2 substrates [2–5]. This pattern is characterized by uniformly distributed DFS in interphasic nuclei, staining of mitotic chromosomes, and reactivity against a 70–75 kDa protein by immunoblotting [6].

Anti-DFS autoantibodies specifically target the nuclear protein DFS70, most commonly known as lens epithelium-derived growth factor p75 (LEDGF/p75) [7–9]. DFS70/LEDGFp75 is a stress response transcription coactivator that protects mammalian cells against diverse environmental stressors [8]. This autoantigen is also essential for the integration of human immunodeficiency virus 1 (HIV-1) [10]. In addition, it has been recognized as an oncoprotein whose overexpression in cancer cells promotes tumor aggressive properties such as increased clonogenicity, migration, invasion, chemotherapy resistance, stress survival, angiogenesis and tumor growth (reviewed in Refs. [8, 9]).

The clinical and biological significance of autoantibodies to DFS70/LEDGFp75 remains enigmatic, although it is plausible that these antibodies arise in response to molecular and cellular events associated with altered structure or expression of this antigen under a pro-inflammatory microenvironment [8]. While these autoantibodies have been reported at relatively low frequencies (<5%) in patients with AARD and inflammatory myopathies (IIM), their prevalence in apparently healthy individuals (HI) and young women appears to be higher [2–4, 8, 11–14]. They have also been detected at varied frequencies in non-rheumatic conditions such as atopic dermatitis, interstitial cystitis, prostate cancer, and eye diseases [3, 4, 6, 8]. When present in patients with AARD, anti-DFS70/LEDGFp75 antibodies are typically accompanied by other ANA, but in HI and patients with non-AARD conditions they tend to be monospecific, hence becoming attractive clinical biomarkers to potentially exclude a diagnosis of AARD [12–17].

Recent studies on anti-DSF70/LEDGFp75 autoantibodies have highlighted inter-laboratory discrepancies in their detection and frequencies, likely due to differences in assay platforms and human expertise in the interpretation of the IIF-DFS ANA pattern, or, possibly, variations in the gender, ethnicity, geographical location, and environmental exposures of the populations screened for the presence of these autoantibodies [4, 8]. While their frequency has been widely examined in populations of European and Asian descent, as well as in Brazilians, their frequency in Hispanic/Latino populations has not been reported. In this study, we examined for the first time the frequency of anti-DSF70/LEDGFp75 autoantibodies in sera from Mexican patients with RA, dermatomyositis (DM), and obesity (OB), as well as in Mexican HI. Multiple complementary assay platforms were used in order to develop consensus regarding the types of techniques/platforms that provide accurate detection and confirmation of these autoantibodies.

## Materials and methods

### Patient sera

Patients diagnosed with RA, DM, and OB were recruited from Hospital Civil de Guadalajara “Juan I. Menchaca”, Centro Medico Nacional 20 de Noviembre, ISSSTE (“Instituto de Seguridad y Servicio Social de los Trabajadores del Estado”), Hospital General de Mexico and Centro Médico Nacional “La Raza”, IMSS (“Instituto Mexicano del Seguro Social”) of Mexico city. Clinical history of serum donors was available. We included a group of apparently healthy adult individuals (HI) defined as non-obese (body mass index 18.0–24.99 kg/m^2^) without history of chronic illness such as hypertension, diabetes mellitus, dyslipidemia, etc. Patient recruitment and serum analysis were conducted under consent and authorization from Institutional Review Boards of Hospital Civil “Dr. Juan I. Menchaca” and Loma Linda University. Serum samples were numerically coded to remove patient identifiers during biological analysis.

### ANA detection

Antinuclear antibody were detected in patient sera as described previously [7]. Briefly, sera were used at 1:80 and 1:160 dilutions, and ANA were visualized by IIF using HEp-2 substrates (NOVA Lite^®^ HEp-2 ANA, Inova Diagnostics, San Diego, CA), following the manufacturer’s instructions. Chromatin was counterstained with 4′,6-diamidino-2-phenylindole (DAPI). Visualization of ANA patterns and chromatin, and image acquisition were done on a Keyence BZ9000 Biorevo fluorescence microscope.

### Enzyme-linked immunosorbent assay (ELISA)

DFS70-ELISA Kit (MBL International Corporation, Woburn, MA) was used to specifically detect anti-DFS70/LEDGF autoantibodies in patient sera. Sera were diluted at 1:100 and analyzed for the presence of these autoantibodies according to manufacturer’s instructions. Optical density (OD) values were obtained at 450 nm in a plate reader. We classified positivity to anti-DFS70/LEDGFp75 by two parameters, using a reference value of ≥15 U/mL (recommended by the kit manufacturer) and mean U/mL plus two standard deviations (mean + 2SD). Each serum sample was tested in duplicate.

### Western blotting

Western blotting (WB) procedures were essentially conducted as previously described [7]. Briefly, SDS-PAGE (NuPAGE 4–12% gels, Life Technologies) was used to separate total cellular proteins from Jurkat T cells (ATCC^®^ TIB-152™) or prostate cancer PC3 cells (ATCC^®^ CRL-1435™), followed by transfer to nitrocellulose membranes (GE Healthcare Life Sciences). Membranes were cut into individual strips and blocked overnight with 5% dry milk solution in TBS-T buffer (20 mM Tris–HCl, pH 7.6, 140 mM NaCl, 0.1% Tween 20) and individual strips were then probed with different patient sera for 2 h. After several washes with TBS-T, the individual membrane strips were incubated for 1 h with horseradish peroxidase (HRP)-conjugated goat anti-human secondary antibody (ThermoFisher Scientific, Cat# A18847, used at 1:5000 in 5% milk/TBS-T). Detection of serum autoantibodies bound to proteins was achieved by enhanced chemiluminescence (ECL) (Thermo Fisher Scientific Pierce) in autoradiography films.

### DFS70 chemiluminescent assay

Sera that exhibited the DFS-IIF pattern and recognized a 70–75 kDa protein band by WB were further analyzed by the QUANTA Flash^®^ DFS70 chemoluminescent assay (CIA) to confirm the presence of anti-DFS70/LEDGFp75 antibodies as described previously [3, 7, 14]. This assay is hereafter referred to as DFS70-CIA and uses recombinant DFS70/LEDGFp75 protein coated onto paramagnetic beads. Briefly, the relative light units (RLUs) were proportional to the amount of isoluminol conjugate bound to the anti-human IgG, which in turn was proportional to the amount of serum autoantibodies bound to recombinant DFS70/LEDGFp75 immobilized in the beads. Using a standard curve, RLU values were converted into calculated units (CU). Sera with CUs less than 20 were considered negative for DFS70/LEDGFp75 antibodies while those with CUs 20–100, 101–300, and >300 were considered low positive, moderately positive, and high positive, respectively.

## Results

### Prevalence of anti-DFS70/LEDGFp75 autoantibodies as detected by ANA-IIF

**Fig. 1:**
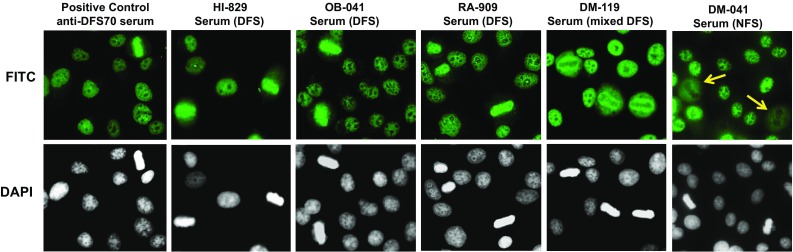
Detection of the dense fine speckled (DFS) immunofluorescent pattern using human sera. Representative human sera displaying the characteristic DFS nuclear pattern in HEp-2-ANA slides, visualized by IIF microscopy using FITC-labeled secondary antibodies, are shown in the first five panels from *left* to *right*. Corresponding DAPI staining is shown in *black* and *white* for better visualization of chromatin. Note that the mitotic chromatin is brightly stained with the positive control sera and with sera HI-829, OB-041, and RA-909, but the DFS staining pattern produced by serum DM-119 was atypical since the mitotic chromatin was stained only at the edges. Serum DM-041 in the far *right panel* displays the characteristic NFS pattern (unrelated to the DFS pattern). *Yellow arrows* point to the negative staining of mitotic chromatin

We included in this study 71 patients with DM, 47 patients with RA, 30 patients with OB, and 23 HI. All patients and HI were Mexican Hispanics living in Mexico. Serum samples from these groups were evaluated by ANA-IIF assay for the presence of the classical dense fine speckled (DFS) pattern characterized by nuclear dense fine speckles, often excluding the nucleoli, with staining of mitotic chromosomes [6, 7]. Other ANA patterns were also recorded in the analysis. Table 1 shows a relatively low frequency of the DFS pattern in the three disease groups tested (1.4% in DM, 4.3% in RA, 6.7% in OB), but higher in HI (17.4%). It should be noted, however, that only one of the 4 sera from HI (HI-829) that tested positive for the DFS pattern showed a strong reactivity, compared to a positive control serum, at 1:160 dilution (Fig. 1). The other HI sera showed relatively weak staining at this dilution (data not shown). Other sera that showed strong anti-DFS immunoreactivity at 1:160 dilution included OB-041 and RA-909 (Fig. 1). Another serum, DM-119, displayed DFS staining in interphasic nuclei, but the mitotic chromatin staining was concentrated at the edges of the metaphase plate (Fig. 1).

**Table 1:** Frequency of serum antibodies recognizing the dense fine speckled and the nuclear fine speckled patterns by ANA-IIF assay

ANA pattern	DM (*n* = 71)	RA (*n* = 47)	Obesity (*n* = 30)	HI (*n* = 23)
DFS (mc +)	1 (1.4)	2 (4.3)	2 (6.7)	4 (17.4)
NFS (mc −)	31 (43.7)	24 (51.1)	5 (16.7)	3 (13.0)
Other patterns	9 (12.7)	17 (36.2)	4 (13.3)	1 (4.3)
Negative	30 (42.3)	4 (8.5)	19 (63.3)	15 (65.2)

Values are represented as *n* (%)

*ANA* antinuclear antibody, *DFS* dense fine speckled, *DM* dermatomyositis, *HI* healthy individuals, *mc +* mitotic chromatin positive, *mc −* mitotic chromatin negative, *NFS* nuclear fine speckles, *OB* obesity, *RA* rheumatoid arthritis

As indicated in Table 1, the most prevalent ANA-IIF pattern found in the sera, particularly in the DM and RA patient groups, was the nuclear fine speckled (NFS) pattern, which is unrelated to the DFS-IIF pattern. The NFS pattern, defined as the “AC-4 pattern” by the International Consensus on ANA Patterns (ICAP) group, is characterized by tiny fine speckles across the nucleoplasm (excluding or including the nucleoli) with negative staining of mitotic chromatin (http://www.ANApatterns.org) [18]. This pattern can be confused with the DFS pattern if negative staining of the mitotic chromatin is not confirmed. A representative serum producing this pattern is shown in Fig. 1 (serum DM-041). In the DM group, 31 of 71 patients (43.7%) showed the NFS pattern and of these, 11 sera showed weak reactivity at 1:160 dilution. In the RA group, 24 of 47 patients (51.1%) exhibited this pattern, although the majority of these (*n* = 14) showed very weak reactivity at 1:160 dilution. The 5 OB sera and 2 of the 3 HI sera producing this NFS pattern showed weak reactivity at 1:160 dilution.

### Confirmation of anti-DFS70/LEDGFp75 autoantibodies by ELISA and WB

**Fig. 2:**
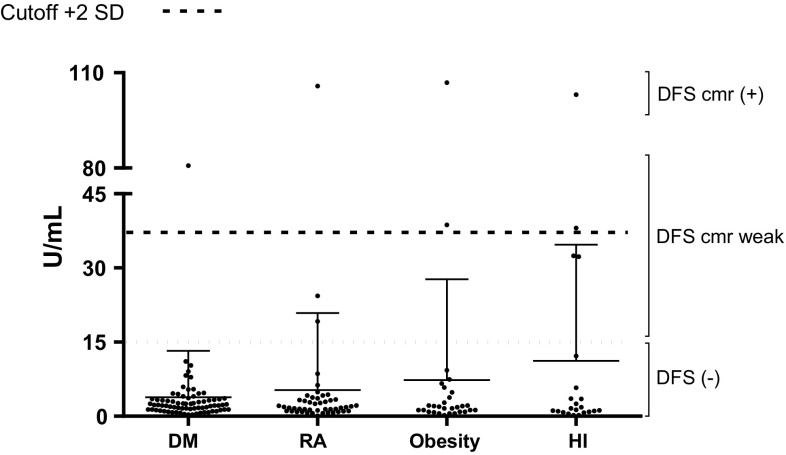
Detection of anti-DFS70/LEDGFp75 antibodies by ELISA. Antibody levels in the different patient groups and healthy individuals are represented as Units/mL. The cut-off represents the mean plus two standard deviations

All the sera from the four groups (*n* = 171) were evaluated for the presence of anti-DFS70/LEDGFp75 autoantibodies using a commercially available DFS70-ELISA kit, with values ranging from 0.10 to 106.87. As shown in Fig. 2 and Table 2, only 10 serum samples were found positive for anti-DFS70/LEDGFp75 autoantibodies when 15 U/mL was taken as a reference value, as recommended by the ELISA kit manufacturer. Of these, 6 sera had values between 19.10 and 38.06, whereas 4 sera had values ranging from 80.76 to 106.87. It should be noted that the ELISA results corresponded to the ANA-IIF results, with the highly reactive positive sera by ELISA (DM-119, OB-041, HI-829, and RA-909) also producing a strong DFS pattern at 1:160 dilution in ANA-IIF assays (Table 3). One exception was serum RA-907, which showed by ANA-IIF a very weak nuclear homogeneous staining at 1:80 dilution, with negative reactivity at 1:160 dilution, but was reactive by ELISA, albeit weakly (OD = 24.38). The serum with the weakest positive ELISA reactivity (RA-880) also showed very weak reactivity by ANA-IIF and did not produce a clearly defined DFS pattern at 1:80 or 160 dilutions (Table 3 and data not shown). The mean ± SD of each group were as follows: DM 3.9 ± 9.39 U/mL, RA 5.3 ± 15.59 U/mL, OB 7.3 ± 20.42 U/mL, and HI 11.2 ± 23.56 U/mL. Interestingly, when we established a cut-off value of mean plus two standard deviations only the top six reactive sera remained positive, DM-119 (80.76 U/mL), RA-909 (105.79 U/mL), OB-156 and OB-0.041 (38.72 and 106.87 U/mL, respectively) and HI-840 and HI-829 (38.06 and 103.10 U/mL, respectively).

**Table 2:** Frequency of anti-DSF70/LEDGFp75 antibodies detected by ELISA according to ANA-IIF assay

Anti-DFS70/LEDGFp75						
	≤2 SD (*n* = 165)	>2 SD (*n* = 6)	*P*	≤15 U/mL (*n* = 161)	>15 U/mL (*n* = 10)	*P*
ANA						
(−)	68 (41.2)	1 (16.7)	0.40	68 (42.2)	1 (10.0)	<0.01
(+)	97 (58.8)	5 (83.3)		93 (57.8)	9 (90.0)	
DFS pattern						
DFS	3 (1.8)	6 (100.0)	<0.01	0 (0.0)	9 (90.0)	<0.01
NFS	63 (38.2)	0 (0.0)		63 (39.1)	0 (0.0)	
Other	31 (18.8)	0 (0.0)		30 (18.6)	1 (10.0)	
Negative	68 (41.2)	0 (0.0)		68 (42.2)	0 (0.0)	

Values are represented as *n* (%), comparisons were performed with *χ* ^2^ or Fisher’s exact tests

*DFS70* dense fine speckles 70 kDa protein, *LEDGF* lens epithelium-derived growth factor, *ANA* antinuclear antibodies, *DFS* dense fine speckled, *NFS* nuclear fine speckled

**Table 3:** Concordance between different assay platforms for the detection of anti-DFS70/LEDGFp75 autoantibodies in selected ANA-positive human sera

Serum	ANA pattern (1:160 dilution)	DFS70-ELISA (cut-off = OD >15 U/mL, 2 SD)	WB (reactivity with 70–75 kDa band)	DFS70-CIA (cut-off = 20 CU)
DM-03	NFS (mc −)	2.63	Negative	1.5
DM-037	Weak NFS (mc −)	1.60	Negative	<1.0
DM-041	Weak NFS (mc −)	3.21	Negative	<1.0
DM-095	Weak NFS (mc −)	3.09	Negative	<1.0
DM-104	Weak NFS (mc −)	2.41	Negative	<1.0
DM-105	Weak NFS (mc −)	3.32	Negative	1.5
DM-119	Strong DFS mixed	80.76	Strong positive with other multiple reactive bands	265.3
DM-156	Weak NFS (mc −)	2.29	Negative	<1.0
RA-860	NFS (mc −)	1.80	Negative	<1.0
RA-862	Strong NFS (mc −)	1.70	Negative	1.2
RA-866	Weak NFS (mc −)	3.10	Negative	<1.0
RA-869	Weak NFS (mc −)	1.30	Negative	<1.0
RA-880	Weak DFS	19.18	Weak positive	23.0
RA-895	Weak NFS (mc −)	2.90	Negative	<1.0
RA-903	Weak NFS (mc −)	1.90	Negative	<1.0
RA-907	Very weak nuclear homogeneous	24.38	ND	ND
RA-909	Strong DFS	105.79	Strong positive	123.8
RA-912	Nuclear coarse speckled	0.60	Negative	<1.0
OB-033	Weak NFS (mc −)	0.92	Negative	<1.0
OB-041	Strong DFS	106.87	Strong positive	166.2
OB-101	Weak NFS (mc −)	0.92	Negative	<1.0
OB-156	Weak NFS (mc −)	38.72	Weak positive	72.8
OB-163	Weak NFS (mc −)	0.80	Negative	<1.0
OB-165	Weak homogeneous	5.84	Negative	1.2
HI-808	Weak DFS	32.27	Weak positive	46.3
HI-817	Weak DFS	32.47	Moderately strong positive	45.9
HI-820	NFS (mc −)	0.50	Negative	<1.0
HI-825	Weak NFS (mc −)	1.30	Negative	<1.0
HI-829	Strong DFS	103.10	Strong positive	308.1
HI-840	Weak DFS	38.06	Weak positive	24.5
HI-847	Weak NFS (mc −)	1.60	Negative	<1.0
HI-849	Weak NFS (mc −)	2.00	Negative	<1.0

*ANA* antinuclear antibody, *DFS* dense fine speckled, *DFS70* dense fine speckled autoantigen of 70 kDa, *CIA* chemiluminescent assay, *CU* chemiluminescent units, *DM* dermatomyositis, *ELISA* enzyme-linked immunosorbent assay, *HI* healthy individuals, *mc −* mitotic chromatin negative, *NFS* nuclear fine speckles, *ND* not determined, *OB* obesity, *OD* optical density, *RA* rheumatoid arthritis, *SD* standard deviation, *WB* Western blotting

**Fig. 3:**
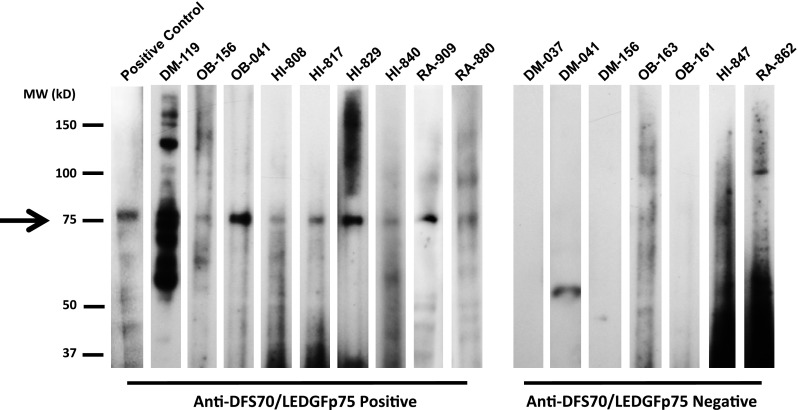
Detection of anti-DFS70/LEDGFp75 antibodies by Western blotting. Immunoblot strips showing the immunoreactivity of representative human anti-DFS (anti-DFS70/LEDGFp75 positive) and anti-NFS (anti-DFS70/LEDGFp75 negative) against whole lysates from Jurkat T cells. *Arrow* points to positive reactivity of anti-DFS70/LEDGFp75 sera against a 75-kDa protein band. Sera OB-041, HI-829, and RA-880 reacted with the highest intensity, whereas serum DM-119 reacted against multiple protein bands, including a 75-kDa band, consistent with its atypical DFS staining pattern observed in Fig. 1

We then selected the sera that had positive anti-DFS70 results by ANA-IIF for confirmation by WB analysis, using whole protein lysates from two cancer cell lines, Jurkat and PC3, which we have previously shown to express elevated levels of the DFS70/LEDGFp75 protein [7, 19, 20]. For comparison, we also tested by WB sera that showed the NFS pattern by ANA-IIF but were negative for anti-DFS70/LEDGFp75 autoantibodies by ELISA. We observed that the sera that were the most highly reactive against DFS70/LEDGFp75 by ANA-IIF and ELISA (DM-119, OB-041, HI-829, and RA-909) also reacted strongly with a protein band of approximately 75 kDa by WB in Jurkat T cell lysates (Fig. 3; Table 3). Weakly reactive sera by ANA-IIF and ELISA also showed very weak reactivity by WB, which was visualized only after film overexposure to ECL reagent. Serum RA-907, which showed by ANA-IIF a very weak nuclear homogeneous staining but reacted weakly by ELISA, gave negative reactivity against a 70–75 kDa protein by WB (data not shown). These results were reproduced in PC3 cell lysates (data not shown). WB analysis of selected sera that produced the NFS pattern and gave negative results in DFS70-ELISA did not reveal reactivity against protein bands in the 70–75 kDa region (Fig. 3).

### Confirmation of DFS70/LEDGFp75-positive sera by DFS70-CIA

As final confirmation of the presence of anti-DFS70/LEDGFp75 antibodies in sera, we analyzed by DFS70-CIA the 9 sera that tested positive for these antibodies by the three assays, ANA-IIF, ELISA, and WB. We also included in the analysis 22 selected sera (7 DM, 7 RA, 4 OB, and 4 HI) that showed the NFS pattern by ANA-IIF and were negative for anti-DFS70/LEDGFp75 antibodies in ELISA and WB. Consistent with the above results, all 9 sera that produced the classical anti-DFS ANA-IIF pattern and reacted positively against DFS70/LEDGFp75 by ELISA and WB also gave positive results by DFS70-CIA, with high concordance between the DFS70-CIA and ELISA values (Table 3). As expected, sera producing the NFS pattern with negative reactivity against DFS70/LEDGFp75 by ELISA and WB were also negative by DFS70-CIA.

## Discussion

The presence of anti-DFS70/LEDGFp75 autoantibodies in apparently HI and in a broad spectrum of non-AARD inflammatory conditions have made difficult to pinpoint their clinical and biological significance [4, 5, 8]. Thus far, the strongest associations found for these antibodies are younger age and female gender (reviewed in Ref. [4]), but these results need to be confirmed with very large cohorts from different geographic locations and standardized antibody detection methods. There is also evidence suggesting that the relatively low frequency (<5%) of these antibodies, particularly when they are the sole ANA pattern in serum, in AARD makes them potentially useful biomarkers to rule out the presence of systemic autoimmune disease, although they may not help in ruling out RA [4, 14–17].

It is still unclear what the presence of these autoantibodies is telling us [4, 8]. Our group proposed recently that depending on the context in which they arise these antibodies could play protective or pathogenic roles, or serve as “sensors” or “reporters” of increased oxidative stress or inflammatory cellular damage associated with DFS70/LEDGFp75 upregulation or proteolytic cleavage [8]. Given the enigmatic nature of anti-DFS70/LEDGFp75 autoantibodies, it was also emphasized that when assessing their frequency in different populations, investigators and clinicians should carefully consider the individual’s health history, ethnicity, geographic location, lifestyle, and exposure to environmental stressors [8].

While the frequency and properties of anti-DFS70/LEDGFp75 autoantibodies have been widely investigated in European, Caucasian American, European, Brazilian, and Asian populations [4, 8], to our knowledge their frequency has not been determined in relatively homogeneous Hispanic/Latino populations with and without AARD. The present study was designed to determine whether the reported low frequencies of these autoantibodies in rheumatic diseases and relatively elevated frequencies in HI can be also reproduced in a Hispanic Mexican cohort that included healthy individuals as well as patients with obesity, DM, and RA. To ensure the accurate detection of these autoantibodies, we initially tested for their presence using the HEp-2 based ANA-IIF test and then confirmed the results using three different but complementary detection platforms. These confirmatory tests were necessary in light of growing concerns that the recognition of the DFS pattern by the ANA-IIF test still remains challenging in the clinical laboratory setting [7, 21–25].

Recently, it was emphasized at the 2nd ICAP Workshop and the 2nd International Autoantibody Standardization (IAS) Workshop, both conducted in conjunction with the 12th Dresden Symposium on Autoantibodies, that the DFS-IIF pattern, defined as the “AC-2 pattern” by the ICAP, should be carefully differentiated from the homogenous as well as other speckled nuclear patterns, including the NFS pattern [26]. Conrad and colleagues [26] pointed that the identification of the DFS pattern by HEp-2 IIF is not sufficient for accurate detection of anti-DFS70/LEDGFp75 autoantibodies, and confirmatory assays such as CIA and ELISA, or HEp-2 IIF with serum pre-adsorption using recombinant DFS70/LEDGFp75 protein, are required. Routine confirmation of the presence of these autoantibodies in the clinical setting is likely to prevent additional unnecessary tests for AARD diagnosis, resulting in cost-effective patient management. For instance, a recent study by Gundín et al. [27] conducted in Spain implemented a new workup ANA algorithm that included testing for the presence of anti-DFS70/LEDGFp75 antibodies, using confirmatory assays, to clinically discriminate AARD from non-AARD patients in ANA-IIF positive individuals, resulting in significant cost-effective patient management in their setting.

In our initial HEp-2 IIF analysis, we found several sera that produced a nuclear staining pattern consistent with the description of the NFS pattern. It was therefore necessary to distinguish these NFS sera from the DFS sera by conducting confirmatory tests to validate the presence of autoantibodies to DFS70/LEDGFp75 in the DFS sera and their absence in the NFS sera. We found a strong concordance in the detection of anti-DFS70/LEDGFp75 by the different confirmatory tests in the DFS sera, highlighting the growing consensus that confirmation of a positive DFS pattern by the ANA IIF-HEp-2 test should be adequately achieved with additional tests [4, 5, 7, 21, 22, 24, 25]. However, we suggest that confirmatory WB data should be interpreted with caution given that whole lysates prepared from different cell lines may have varying expression levels of the DFS70/LEDGFp75 protein [19, 20], which could lead to inter-laboratory variations in DFS serum reactivity if the same cell line is not universally used. In our experience, cancer cell lines such as Jurkat, PC3, DU145, HeLa (including its derivative HEp-2), HCT116, and U2OS express elevated levels of DFS70/LEDGFp75, compared to non-cancer or non-transformed cell lines, thus providing excellent sources of this antigen for recognition by high titer anti-DFS antibodies [19, 20]. A challenge that we encountered in our study, however, was that low titer anti-DFS sera reacted very weakly against a 70–75 kDa protein band by WB in both Jurkat and PC3 cells, and often with high background. An alternative to using whole cell lysates for WB detection of anti-DFS antibodies, is to confirm the DFS-IIF pattern by dot blot or line blot methods that use the full length DFS70/LEDGFp75 or a truncated C-terminal fragment of this protein containing the autoepitope region [22].

In our study, the DFS70-ELISA (MBL) and DFS70-CIA (Inova) platforms showed excellent detection sensitivity and concordance between the anti-DFS values obtained, in spite of the differences in the DFS70/LEDGFp75 antigens used, which is consistent with previous results [14]. Some studies have reported a high frequency (>15%) of anti-DFS70/LEDGFp75 antibodies detected by ELISA (non-MBL) in healthy controls as well as in various non-AARD disease conditions [6, 28–30]. It should be noted that in many cases these high frequencies have not been confirmed independently using DFS70-CIA or ELISA-MBL, suggesting that the source and form of the DFS70/LEDGFp75 antigen used, type of equipment or assay used for detection, and the level of stringency used to calculate the cut-off values may influence these frequencies.

The relatively low frequency of anti-DFS70/LEDGFp75 autoantibodies in the disease groups that we tested by the different assay platforms (1.4% in DM, 4.3% in RA) is consistent with previous frequencies reported in the literature for rheumatic diseases [4, 8]. The 6.7% frequency found in OB patients is higher than that typically found in patients with AARD (<5%), but close to the average for HI (6.45% average from 14 studies, range 0–21.6%) [4]. Consistent with previous studies in other ethnic populations, we observed the highest frequency in apparently HI (17.4%); however, this value is close to the top of the 0–21.6% range reported in the literature [4]. These results suggest that geographic differences or ethnicity may not influence the frequency of these autoantibodies. However, given that our sample size for apparently HI (*n* = 23) was relatively small, further studies with larger cohorts of healthy Mexicans are needed to determine if the frequencies of anti-DFS autoantibodies, detected by several confirmatory assays, are indeed higher than those previously reported in HI from other ethnic groups. If this turns out to be true, then it would be of interest to determine if there are any unique genetic, lifestyle, environmental, or inflammatory disease-related factors in Mexican and other Hispanic/Latino populations that might be associated with these elevated frequencies.

## Abbreviations

AIDS: Acquired immunodeficiency syndrome
ANA: Antinuclear antibody
AARD: ANA-associated rheumatic diseases
CIA: Chemiluminescence assay
DFS: Dense fine speckled
DM: Dermatomyositis
ELISA: Enzyme-linked immunosorbent assay
HI: Healthy individuals
HIV: Human immunodeficiency virus
ICAP: International consensus on ANA patterns
IIF: Indirect immunofluorescence
LEDGF: Lens epithelium-derived growth factor
NFS: Nuclear fine speckled
OB: Obesity
RA: Rheumatoid arthritis
WB: Western blotting
